# Western Countries Have Shown Poorer Rather Than Better Performance Against COVID-19

**DOI:** 10.1007/s44229-022-00022-x

**Published:** 2022-11-30

**Authors:** Alberto Boretti

**Affiliations:** Wellington, New Zealand

**Keywords:** COVID-19, Epidemiology, Public health

## Abstract

Western countries have been subjectively claimed to have performed much better than other countries in combatting COVID-19 because of their alleged democratic governance. Here, I show that the performance of their pharmaceutical and non-pharmaceutical measures has in fact been poorer than that in non-Western countries, not because of democratic governance but only because of poor decision-making.

## To the Editor

Jain et al. [1] unexpectedly claim that what they believe is democratic governance might have played an important role in mitigating the overall health impact of COVID-19 across countries. They subjectively praise the pharmaceutical and non-pharmaceutical measures implemented by Western countries, such as the United Kingdom or the United States. In fact, these countries, as compared with many other countries, have performed very poorly in the fight against COVID-19, on the basis of objective criteria [2], regardless of whether their decision-making has been attributed to more or less democracy.

**Fig. 1:**
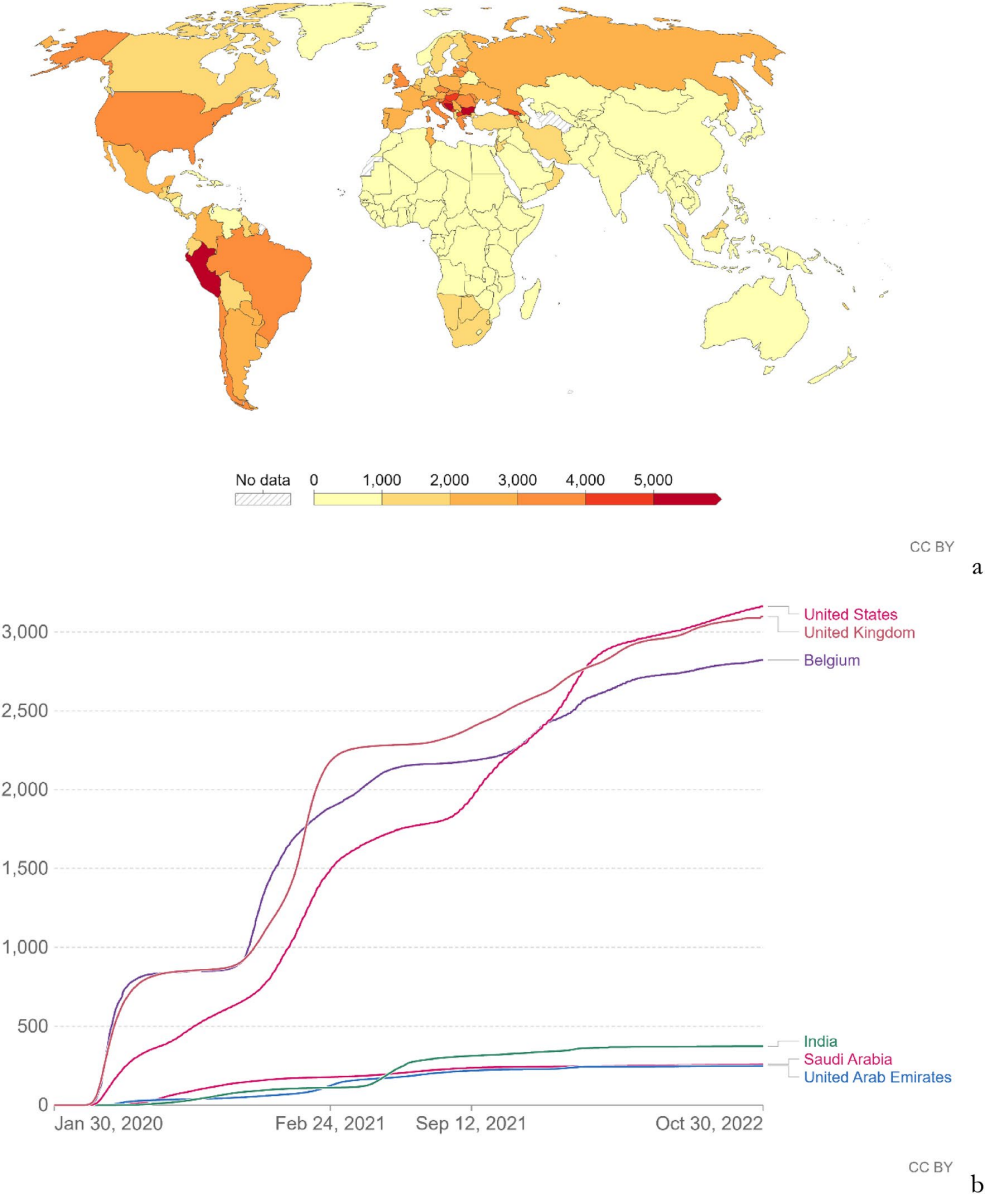
**a** Map of cumulative COVID-19 fatalities per million people (to November 9, 2022). Image from [2]. CC BY. **b** Time series of cumulative COVID-19 fatalities per million people in the United Kingdom, United States, Belgium, India, Saudi Arabia, and the United Arab Emirates. Image from [2]. CC BY

If we consider the total number of COVID-19 fatalities per million people (Fig. 1), which should be objective criteria for assessing the efficacy of pharmaceutical and non-pharmaceutical measures, the United Kingdom and the United States had 3070 and 3135 fatalities per million people, respectively [2]. Their performance was much worse than that of countries with comparable gross domestic product (GDP) per capita, such as the United Arab Emirates or Saudi Arabia, which only had 250 and 260 fatalities per million people, respectively [2], or roughly 15 times fewer fatalities. In addition, countries such as India, despite their much lower GDP per capita, and more challenging societal and medical conditions than those in Western countries, showed enormously better performance, at 375 fatalities per million people [2]. Figure 1 also clearly shows that Africa and Asia outperformed Europe and North/South America. The low fatalities in Australia/New Zealand in Oceania resulted from extreme restrictions, such as the prohibition of citizens of Australia from exiting and in practice entering the country until November 2021 [5], when the much more infective but less lethal Omicron variant emerged and displaced the prior variants.

In conclusion, there is absolutely nothing to worship in the pharmaceutical and non-pharmaceutical measures used by Western countries, such as the United Kingdom and the United States, against COVID-19. These measures were often very difficult to explain with clinical [3] or epidemiological arguments [4], and they did not result from more democratic or scientific processes than those followed by other countries. Moreover, that countries such as the United Arab Emirates, Saudi Arabia, and India performed much better than countries such as Belgium, the United Kingdom, and the United States should be acknowledged.

The markedly lower number of fatalities per million people in non-Western countries was the result of a more genuine attempt to address a medical emergency by listening to medical advice, and using better pharmaceutical and non-pharmaceutical measures to minimize the impact of COVID-19, rather than to focus on gaining political advantages. For example, the United Arab Emirates, Saudi Arabia, and India used every possible antiviral agent demonstrating efficacy against COVID-19, particularly in the early stages of the pandemic, to prevent more serious consequences of infection, whereas Belgium, the United States, and the United Kingdom drastically limited the freedom of medical physicians to prescribe drugs, thus practically leaving most patients untreated [6].

Although no treatment, vaccine, or intervention was fully available and effective in all cases, the use of all practical, effective, and safe modalities based on an objective risk/benefit analysis by experts returned better results. The alleged democratic governance of Western countries mitigating the overall health impact of COVID-19 described in reference [1] therefore conflicts with evidence. The performance of pharmaceutical and non-pharmaceutical measures has been worse rather than better in Western countries, not because of democratic governance but only because of poor decision-making.

## Data Availability

Data are available at the listed source.

## Abbreviation

GDP: Gross domestic product
